# Oncological Outcomes of Non-Urothelial Bladder Cancers in a Specialized Cancer Hospital of a Developing Country

**DOI:** 10.7759/cureus.9957

**Published:** 2020-08-23

**Authors:** Siddique Adnan, Muhammad Arshad Irshad Khalil, Shaukat Fiaz, Muhammad Abu Bakar, Azfar Ali, Zubair Ahmad Cheema, Khurram Mir

**Affiliations:** 1 Surgical Oncology, Shaukat Khanum Memorial Cancer Hospital and Research Centre, Lahore, PAK; 2 Urology, Institute of Kidney Diseases, Peshawar, PAK; 3 Biostatistics and Epidemiology, Shaukat Khanum Memorial Cancer Hospital and Research Centre, Lahore, PAK

**Keywords:** non-urothelial bladder cancer, radical cystectomy, chemotherapy

## Abstract

Background

Non-urothelial bladder cancers (NUBCs) constitute only 5% of all bladder cancers. Because of the scarcity of data, no standardized treatment can be offered to these patients. Surgical treatment can be offered to patients with localized disease; however, generally, the prognosis is unfavorable.

Methodology

Patients with histology-proven NUBC presenting to the Department of Uro-oncology, Shaukat Khanum Memorial Cancer Hospital and Research Center, Lahore, Pakistan, from January 2002 to July 2017 were identified and assessed retrospectively.

Results

A total of 20 patients with a median presenting age of 52 years (range: 34-87 years) were identified. Clinically, T3 was the commonest stage of presentation, i.e., in 11 (55%), whereas 1 (7.1%) patient had metastatic disease. Four types of NUBCs were identified: adenocarcinoma, squamous cell carcinoma, small cell carcinoma, and inflammatory myofibroblastic tumor. Most of the patients with adenocarcinoma were offered surgical treatment in the form of either partial (64.3%) or radical (28.6%) cystectomy. Two patients with small cell carcinoma and two of the three patients with SCC could only be offered palliative chemotherapy. During a median follow-up of 40 months, 14 (70%) patients developed disease progression or recurrence. All these patients succumbed to their disease during a median period of 37.5 months (range: 5-84 months). Furthermore, three- and five-year disease-free survival was 60% and 51%, respectively, and overall survival was 65% and 31%, respectively.

Conclusions

NUBC is a rare but aggressive disease that presents at an advanced stage in many cases. Treatment protocols are not uniform; therefore, further collaborative research is needed to improve survival outcomes.

## Introduction

Bladder cancer (BC) is the 7th most common cancer in men and 17th most common malignancy in women [1]. In 90-95% of patients, BC is of urothelial origin [2], known as transitional cell carcinoma (TCC), and the rest small percentage is composed of non-urothelial BCs (NUBCs). Like TCC, these uncommon cancers exhibit geographical variations, e.g., regions with endemic schistosomal cystitis show a higher incidence of squamous cell carcinoma (SCC) [3]. Surgery can provide cure in localized cancers, whereas palliative chemotherapy or radiotherapy remain the mainstay of treatment in non-resectable and metastatic cancers [4]. These cancers can also present in combination with urothelial cancers as mixed histologies. All mixed or pure NUBCs are considered high-risk disease and generally have a poor prognosis as compare to patients with TCCs, and patients usually present at an advanced stage [5]. Due to these limitations in providing uniform treatment protocols for patients with NUBCs, a continuous research is required on this subject [6]. Here we share our experience in the treatment of these patients at a tertiary care referral cancer center in Pakistan.

## Materials and methods

Data of all patients presenting with BC to the department of Uro-oncology at Shaukat Khanum Memorial Cancer Hospital and Research Center, Lahore, Pakistan, from January 2002 to July 2017 were reviewed retrospectively, and all adult patients between 18 and 87 years of age with histologically proven NUBCs were included in the study. Patients were followed for a median duration of 40 months (range: 5-92 months). Statistical analysis was conducted using IBM SPSS Statistics for Windows, Version 20.0 (IBM Corp., Armonk, NY, USA). The Kaplan-Meier method was used to estimate disease-free survival (DFS) and overall survival (OS). Formal approval was obtained from the Institutional Review Board before commencing data collection.

## Results

A total of 20 patients were found to have NUBCs in the study duration. Out of them, 14 patients had adenocarcinoma, 3 had SCC, 3 had small cell carcinoma, and 1 had inflammatory myofibroblastic cancer. The median presenting age of the patients was 52 years (range: 34-87 years), and hematuria was the commonest presenting concern, i.e., in 17 (85%) patients. Three patients presented with lower urinary tract symptoms. Clinically, T3 was the commonest stage of presentation, and one patient had metastatic disease (Table 1).

**Table 1:** Clinical disease staging SCC, squamous cell carcinoma; IMT, inflammatory myofibroblastic tumor; cT, clinical primary tumor; cN, clinical lymph nodes; cM, clinical metastasis; T2, tumor invades detrusor muscle; T3, tumor invades perivesical tissue; T4, tumor invades any of the following: prostate stroma, seminal vesicles, uterus, vagina, pelvic wall, abdominal wall; N0, no regional lymph node metastasis; N1, metastasis in a single lymph node in the true pelvis (hypogastric, obturator, external iliac, or presacral); M0, no distant metastasis; M1, distant metastases

Variables	Stages	Adenocarcinoma, 14 (70.0%)	SCC, 3 (15.0%)	Small cell carcinoma, 2 (10.0%)	IMT, 1 (5.0%)
cT	T2	4 (28.6)	-	-	-
	T3	7 (50.0)	3 (100.0)	-	1 (100.0)
	T4	3 (21.4)	-	2 (100.0)	-
cN	N0	11 (78.6)	3 (100.0)	1 (50.0)	0 (0.0)
	N1	3 (21.4)	-	1 (50.0)	1 (100.0)
cM	M0	13 (92.9)	3 (100.0)	2 (100.0)	1 (100.0)
	M1	1 (7.1)	-	-	-

The pathological staging according to tumor histologies is presented in Table 2.

**Table 2:** Pathological disease staging SCC, squamous cell carcinoma; IMT, inflammatory myofibroblastic tumor; pT, pathological primary tumor; pN, pathological primary lymph nodes; Tx, primary tumor cannot be assessed; T1, tumor invades subepithelial connective tissue; T2, tumor invades detrusor muscle; T3, tumor invades perivesical tissue; T4, tumor invades any of the following: prostate stroma, seminal vesicles, uterus, vagina, pelvic wall, abdominal wall; Nx, regional lymph nodes cannot be assessed; N0, no regional lymph node metastasis; N1, metastasis in a single lymph node in the true pelvis (hypogastric, obturator, external iliac, or presacral); N2, metastasis in multiple regional lymph nodes in the true pelvis (hypogastric, obturator, external iliac, or presacral)

Variables	Stages	Adenocarcinoma, 14 (70.0%)	SCC, 3 (15.0%)	Small cell carcinoma, 2 (10-.0%)	IMT, 1 (5.0%)
pT	Tx	2 (14.3)	1 (33.3)	2 (100.0)	-
	T1	-	-	-	-
	T2	6 (42.9)	1 (33.3)	-	1 (100.0)
	T3	4 (28.6)	-	-	-
	T4	2 (14.3)	1 (33.3)	-	-
pN	Nx	2 (14.3)	1 (33.3)	2 (100.0)	-
	No	10 (71.4)	1 (33.3)	-	1 (100.0)
	N1	1 (7.1)	1 (33.3)	-	-
	N2	1 (7.1)	-		-

Table 3 demonstrates the treatment patients received. It shows that patients with adenocarcinoma, as they presented at an earlier stage, could be offered curative surgical treatment in most instances. For patients with SCC, one out of three was able to undergo radical cystectomy with adjuvant radiotherapy cover, and the rest two could only be offered palliative chemotherapy. Patients with small cell carcinoma presented with T4 at the outset and received chemotherapy only.

**Table 3:** Treatment modalities with respect to histology SCC, squamous cell carcinoma; IMT, inflammatory myofibroblastic tumor; TURBT, trans-urethral resection of bladder tumor

Variables	Categories	Adenocarcinoma, n = 14	SCC, n = 3	Small cell carcinoma, n = 2	IMT, n = 1
Chemotherapy	None	11 (78.5)	1 (33.3)	-	1 (100.0)
	Adjuvant	2 (14.3)	-	-	-
	Definitive	-	-		-
	Palliative	1 (7.1)	2 (66.7)	2 (100.0)	-
Radiotherapy	None	13 (92.9)	2 (66.7)	2 (100.0)	1 (100.0)
	Adjuvant	-	1 (33.3)	-	-
	Radical	-	-	-	-
	Palliative	1 (7.1)	-	-	-
Surgical procedure	TURBT	1 (7.1)	2 (66.7)	2 (100.0)	-
	Partial cystectomy	9 (64.3)	-	-	1 (100.0)
	Radical cystectomy	4 (28.6)	1 (33.3)	-	-

**Figure 1:**
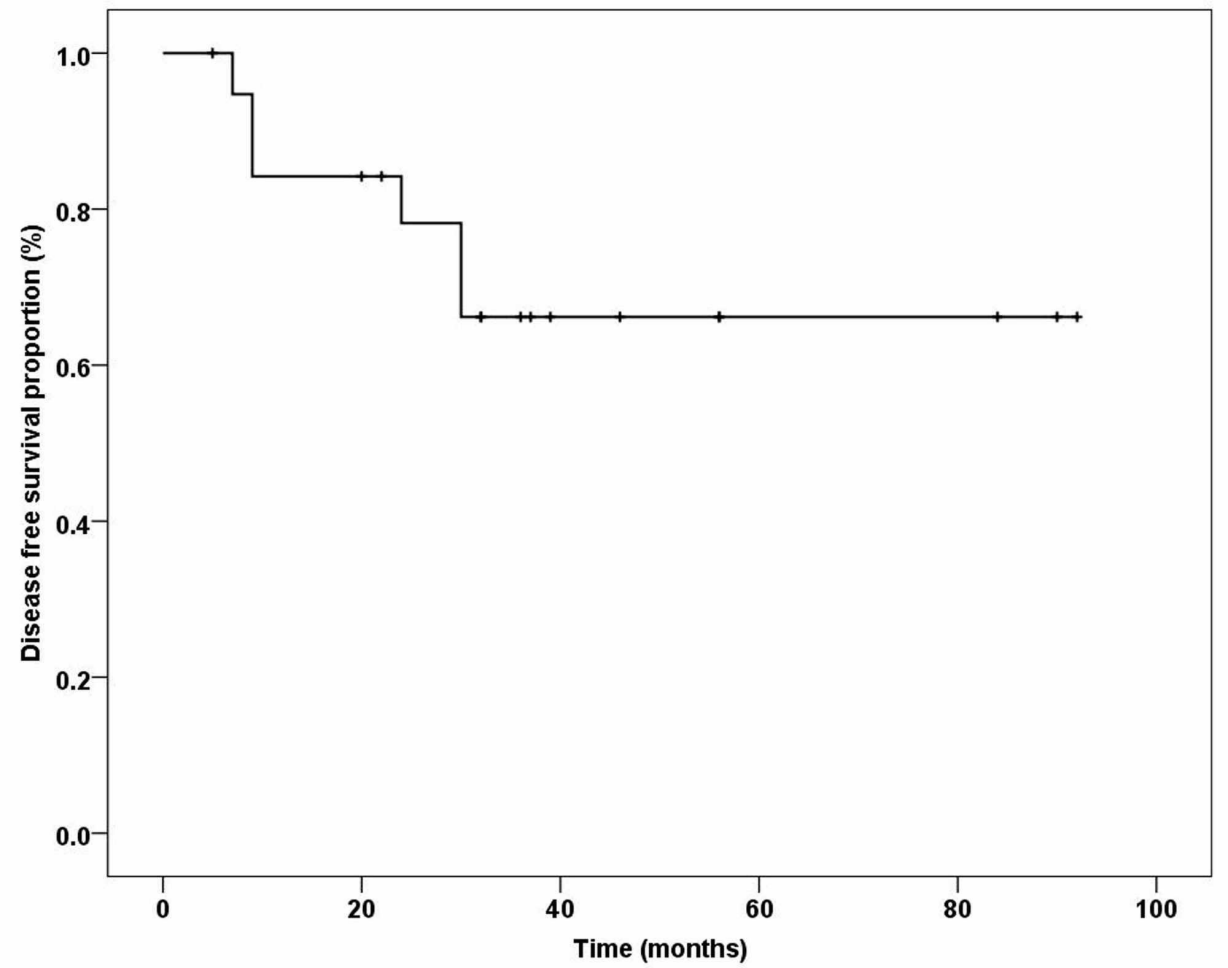
Disease-free survival for patients with non-urothelial bladder cancer (n = 20)

**Figure 2:**
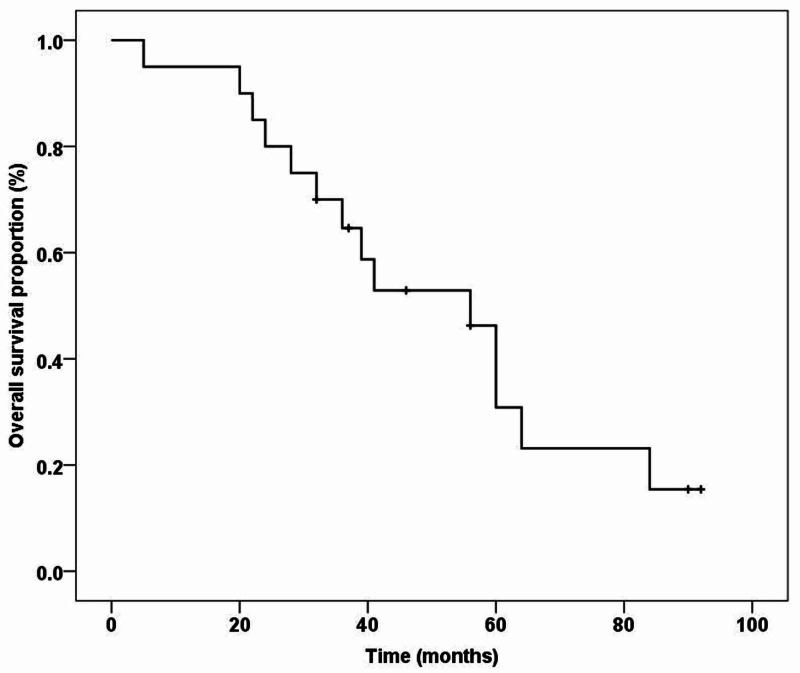
Overall survival with non-urothelial bladder cancer (n = 20)

The median follow-up duration was 40 months (range: 5-92 months). During this period, 14 (70%) patients were diagnosed with cancer recurrence or progression, with majority, i.e., 4 (20%), exhibiting disease at multiple sites. Furthermore, all these patients died because of the disease progression itself (10 patients), and the remaining (4 patients) died of generalized ill health and comorbidities. On analysis, the mean DFS was 59.10 ± 8.66 months (Figure 1), and the overall median survival was 56.0 ± 8.94 months (Figure 2). Furthermore, three- and five-year DFS was 60% and 51%, respectively, and OS was 65% and 31%, respectively.

## Discussion

NUBC is an aggressive disease, and early diagnosis and commencement of treatment are the main factors determining clinical outcomes [7]. Worldwide, SCC is reported to be the most common NUBC, accounting for 3-5% of all BCs [8]. It is most common in Egypt and other African countries, where it is believed to be associated with chronic infection with Schistosoma haematobium [9]. Radical cystectomy is the treatment of choice for localized SCC because benefits from radiation are uncertain, and standard chemotherapy has little effect due to relative chemoresistance of disease [10]. Adenocarcinoma accounts for about 2% of BCs, and to establish the diagnosis of primary adenocarcinoma of the bladder, other possible primary tumors sites such as adenocarcinoma of the prostate or rectum should be ruled out [11,12]. Apart from endemic areas, adenocarcinoma is the most prevalent NUBC. Adenocarcinoma accounted for a major share of NUBC in our study. Standard treatment of all surgically resectable vesical adenocarcinomas consists of radical cystectomy and pelvic node dissection [13].

Small cell carcinoma is much rarer, accounting for less than 1% cases. It occurs mostly during the seventh and eight decades of life. Small cell carcinoma patients with localized disease should be managed radical cystectomy or multimodal therapy including surgery along with chemoradiation. Palliative chemotherapy is reserved for unresectable disease [14]. It carries poor prognosis due to its rarity and aggressive nature [15]. Accordingly, the two patients in our study presented with locally advanced unresectable disease at the outset and received palliative chemotherapy. Both of them died due to disease progression at 20 and 34 months follow-up. One of our patients was proven to have inflammatory myofibroblastic tumor. It is a rare NUBC of the bladder with an unknown possibility of malignant conversion [16]. Treatment strategy comprises transurethral resection, cystectomy, and radiotherapy. Our patient presented at the age of 34 years, was treated with partial cystectomy, and was on regular follow-up for the last 46 months. Additionally, in this study, three- and five-year OS of adenocarcinoma of the bladder was 63% and 27%, respectively. Three-year OS of SCC and adenocarcinoma was 44.8% and 58.7%, respectively [17]. In another study, five-year survival for SCC and adenocarcinoma was reported as 37% and 58%, respectively [16]. The difference in OS at five years of adenocarcinoma was because patients presented with advanced disease stage and relatively small number of sample size as compared to published data.

The median survival of small cell cancer has been reported to be between 10 and 20 months, with 5-20% 5-year OS [18]. The OS of patients diagnosed with SCC and small cell carcinoma cannot be commented due to the small number of participants in our data. Standardized and further collaborative research is needed to move this field forward. Our data also highlight that tools are needed to detect this type of tumors early so that appropriate treatment can be commenced promptly.

## Conclusions

NUBC is a rare disease entity that encompasses a variety of histological subtypes. Our data demonstrate that NUBC is an aggressive disease, which presents with advanced stage and has a poor prognosis. Treatment protocols are not uniform. A combined effort is needed among institutions, both nationally and internationally, to further elucidate the understanding of biology of these tumors and evaluate treatment protocols, which will help in improving long-term survival outcomes.
